# Exposure to low-dose ambient fine particulate matter PM_2.5_ and Alzheimer’s disease, non-Alzheimer’s dementia, and Parkinson’s disease in North Carolina

**DOI:** 10.1371/journal.pone.0253253

**Published:** 2021-07-09

**Authors:** Sung Han Rhew, Julia Kravchenko, H. Kim Lyerly

**Affiliations:** 1 Memory Keepers Medical Discovery Team, University of Minnesota Medical School, Duluth, Minnesota, United States of America; 2 Environmental Health Scholars Program, Division of Surgical Sciences, Department of Surgery, Duke University School of Medicine, Durham, North Carolina, United States of America; 3 Department of Pathology, Duke University School of Medicine, Durham, North Carolina, United States of America; Universidade Federal do Rio Grande - FURG, BRAZIL

## Abstract

Alzheimer’s disease (AD), non-AD dementia, and Parkinson’s disease (PD) are increasingly common in older adults, yet all risk factors for their onset are not fully understood. Consequently, environmental exposures, including air pollution, have been hypothesized to contribute to the etiology of neurodegeneration. Because persistently elevated rates of AD mortality in the southern Piedmont area of North Carolina (NC) have been documented, we studied mortality and hospital admissions for AD, non-AD dementia, and PD in residential populations aged 65+ with long-term exposures to elevated levels of ambient air particulate matter 2.5 (PM_2.5_) exceeding the World Health Organization (WHO) air quality standards (≥10μg/m^3^). Health data were obtained from the State Center for Health Statistics and the Healthcare Cost and Utilization Project. PM_2.5_ levels were obtained from the MODIS/MISR and SeaWiFS datafiles. Residents in the Study group of elevated air particulate matter (87 zip codes with PM_2.5_≥10μg/m^3^) were compared to the residents in the Control group with low levels of air particulate matter (81 zip codes with PM_2.5_≤7.61μg/m^3^), and were found to have higher age-adjusted rates of mortality and hospital admissions for AD, non-AD dementia, and PD, including a most pronounced increase in AD mortality (323/100,000 vs. 257/100,000, respectively). After adjustment for multiple co-factors, the risk of death (odds ratio, or OR) from AD in the Study group (OR = 1.35, 95%CI[1.24–1.48]) was significantly higher than ORs of non-AD dementia or PD (OR = 0.97, 95%CI[0.90–1.04] and OR = 1.13, 95%CI[0.92–1.31]). The OR of hospital admissions was significantly increased only for AD as a primary case of hospitalization (OR = 1.54, 95%CI[1.31–1.82]). Conclusion: NC residents aged 65+ with long-term exposures to ambient PM_2.5_ levels exceeding the WHO standard had significantly increased risks of death and hospital admissions for AD. The effects for non-AD dementia and PD were less pronounced.

## 1. Introduction

Particulates in the air have been associated with poorer human health and lower life expectancy, even at low levels [1]. Based on the World Health Organization (WHO) estimates, 91% of people worldwide live in the areas where levels of particulate matter 2.5 (PM_2.5_, particulate matter with aerodynamic diameter less than 2.5 micrometers) exceed the WHO standards for air quality (a yearly average level of less than 10μg/m^3^ maximum and a 24-hour average of less than 25μg/m^3^ maximum) [1]. Sources of PM_2.5_ include mineral matter, road dust, industry and fuel oil combustion, and toxicity of PM is determined not only by their size and ability to enter an individual’s cardiovascular system by inhalation, but also by their components, including concentration of transition metals (e.g., vanadium, nickel, lead, arsenic, etc.) [2]. Some of these metals can stimulate the generation of hydroxyl radicals in Fenton-type reactions [3–5], resulting in reactive oxygen species (ROS) which damage human cells [6, 7]. In addition to transition metals, organic compounds such as polycyclic aromatic hydrocarbons (PAHs) are associated with aerosol particles from petroleum, coal, wood and biomass burning (coal-derived, motor vehicle-derived, or from other sources) [8]. PAHs has been shown to induce ROS production and decrease antioxidant enzyme activity resulting in oxidative stress in vascular endothelial cells [9], decrease of cardiovascular-related gene expression through upregulating miRNA [10], promotion of carcinogenesis [11] and neuroinflammation and cognitive decline [12, 13]. More recent studies have identified unique toxicological signatures of PM which determine interactions with molecular cascades. Different local spatial and temporal variations of PM levels and different physicochemical properties of the particles’ compounds are associated with different disease-specific outcomes [14]. PM is described as a complex toxin pertaining to diverse central nervous system (CNS) pathology, and the mechanisms of PM affecting the CNS include not only increasing oxidative stress and neuroinflammation, but also causing endoplasmic reticulum (ER) stress, mitochondrial dysfunction, and disturbance of protein homeostasis [14]. As a result, it is now appreciated that the complex dose and time of environmental exposure of individuals to specific metals and toxins in PM can be responsible for inflammation, neurodegeneration, and other chronic disease processes [15].

Understanding the impact of PM on cognitive function and diseases of CNS is critical as widespread reduction in air pollution might significantly reduce the burden of neurodegenerative diseases, among other chronic diseases, in the population [16]. While the risk of PM_2.5_ on respiratory and cardiovascular disease has been previously recognized, there is an increasing number of clinical reports focused on a brain as potential target for PM_2.5_ exposures [14, 17]. The Lancet commissions of 2017 and 2018 included air pollution in a list of potential risk factors for dementia [18] stating that the evidence of causation is increasing, in particular for exposure to fine PM [19]. Although studies on impacts of ambient PM_2.5_ on respiratory and cardiovascular systems have a longer history as compared to studies on the effects on CNS, there is an increasing number of reports focused on a brain as potential target for PM_2.5_ exposures [14, 17] due to its high energy use and high metabolic demand (but more specific features of the central nervous system may also play important roles). The brain has low levels of vitamin C, superoxide dismutase, catalase, and other endogenous scavengers, as well as a high cellular content of lipids and proteins, and extensive axonal and dendritic networks which are vulnerable to oxidative stress damage [20, 21]. Although the underlying mechanism of PM-induced neuroinflammation and neurodegeneration is not fully understood, microglia (the glial cells acting as the primary line of immune defense for the brain) have a critical role in the brain’s inflammatory response by triggering innate immunity [21]. The junction between cerebral microvascular endothelial cells are a structural and functional base of blood-brain barrier (BBB), and a disruption of tight junctions is a key factor in CNS disease leading to deposition of small ambient PM into the brain tissue [22]. Studies of brain of older individuals with a history of long-time exposure to ambient PM demonstrated the presence of aggregation of Aβ42, accumulation of α‐synuclein, and hyperphosphorylation of Tau protein [23], thus suggesting that exposure to PM, plaque formation and neurofibrillary tangles may facilitate pathogenesis of neurodegenerative diseases. Toxicological studies of ambient air pollutants demonstrated induction of oxidative stress and neuroinflammation [22], activation of microglia [24], and stimulation of neural antibodies [25] which could contribute to neurodegeneration.

Alzheimer’s disease (AD) is the most common form of dementia in older adults that affects over 5 million people in the United States (US); by 2050, the number of people living with dementia globally is projected to increase to 152 million [26]. As the US population ages, there is an increasing importance of identifying potentially modifiable risk factors contributing to morbidity and mortality from AD, non-AD dementia, and Parkinson’s disease (PD)–the diseases that impact both patients and their caregivers [27]. While the etiology of AD and other neurodegenerative diseases remains unclear [28, 29], there is growing concern about the role of air contaminants (due to the traffic or other sources of air pollution) in neurodegeneration [30–33] which could result in a higher risk of AD, non-AD dementia or PD [34–36]. Additionally to the growing numbers of laboratory studies, epidemiological evidence for increased neurodegenerative disease risks due to exposure to PM is also growing [37]. Recent studies demonstrated that PM_2.5_ affected pathogenesis of neurodevelopmental disorders and neurodegenerative disease, including autism spectrum disorder [38], AD, non-AD dementia, and PD [27, 32, 36, 39–42]. Reports on an increased risks of AD and non-AD dementias following chronic exposure to PM have come from Taiwan [34], Sweden [32], Spain [43], Germany [44], US [39], Israel [45], and other countries [46]. Recent meta-analyses demonstrated the risk of these diseases increasing with the higher levels of ambient PM_2.5_, as well as PM_10_ and other air contaminants such as NO_2_/NO_x_ and CO [47, 48]. The magnitude of increased risks varies across the studies, depending on populations, geographic area, and considered cofactors.

North Carolina (NC) was ranked number 14^th^ in 2017 among the US states for AD mortality in individuals aged 65+, with persisting high AD mortality in the southern Piedmont area [49]. Socioeconomic characteristics, access to medical care, regulations of recording of vital statistics, multiple comorbidities, and exposure to arsenic (As, which is elevated in the Carolina Slate Belt due to the natural rock characteristics) have been discussed as potential contributors to a higher AD risk; however, these factors were not able to fully explain the observed death patterns [49–53]. The southern Piedmont area attracts older residents with AD who stay in specialized care facilities; however, this factor also cannot explain the observed geographic disparities in AD mortality in NC [49].

Although the average air quality in NC has generally improved over the past twenty years, thus contributing to lower respiratory, cardiovascular, and cerebrovascular mortality [54, 55], there remain geographic areas within the state with the levels of ambient air contaminants consistently exceeding the WHO standards. This study aims to investigate whether long-term exposure of residents to ambient PM_2.5_ impacts mortality and hospital admissions for AD, non-AD dementia, and PD in the southern Piedmont area of NC. We hypothesize that populations exposed to PM_2.5_ levels exceeding the WHO standard (10 μg/m^3^ for maximum yearly average) have higher mortality and hospital admissions for AD, non-AD dementia, and PD compared to populations exposed to PM_2.5_ at the levels below the WHO standard.

## 2. Materials and methods

### 2.1. Data

Data on disease-specific mortality were obtained from the State Center for Health Statistics (SCHS) for 2007–2014 [56]. For the same time period, data on hospital admissions were obtained from the Healthcare Cost and Utilization Project’s (HCUP) State Inpatient Database (SID) [57]. These datasets provide individual level information on diseases, as well as on age and race of each patient. We used zip codes of residential addresses of each individual to perform the analysis of health outcomes, because information on the levels of ambient PM_2.5_, as well as on median household income and education level was available on zip code level. Diseases such as AD (ICD-9 code 331.0, ICD-10 code G30), PD (ICD-9 code 332, ICD-10 code G20), and non-AD dementia (ICD-9 codes 331.1, 331.9, 290, 294.0, 294.10, 294.11, 294.2, 294.21, 294.8, ICD-10 codes G31, F01, F02.80, F02.81, F03) were analyzed in populations aged 65+. These codes were selected in accordance with the Alzheimer’s Association recommendations [58] and the National Center for Chronic Disease Prevention and Health Promotion, Centers for Disease Control and Prevention [59].

#### 2.1.1. Data on exposure to PM_2.5_

Information on ambient PM_2.5_ at zip code level were derived from the National Aeronautics and Space Administration (NASA) data that include annual PM_2.5_ grids from MODerate resolution Imaging Spectroradiometer (MODIS), Multi-angle Imaging SpectroRadiometer (MISR), and Sea-viewing Wide Field-of-view Sensor (SeaWiFS) Aerosol Optical Depth (AOD) measurements. We used the approach developed by Van Donkelaar and the group [60] who combined three satellite information sources on PM_2.5_ measures and adapted a Geographically Weighted Regression (GWR) method to adjust for the residual PM_2.5_ bias in the initial satellite-derived values with 0.01 degrees of resolution (approximately 1.1km). Because federal- and state-funded networks of air quality monitors in the US states are located unevenly and their coverage is sparse in rural areas [61], the reason for choosing the satellite-based data with GWR approach was to extend the data on PM_2.5_ measures to NC regions with sparse locations of PM_2.5_ ground monitors. Previous studies showed that GWR-applied satellite-derived PM_2.5_ concentrations strongly correlated with the values from the US Environmental Protection Agency (EPA) data on PM_2.5_ compliance network (the correlation coefficient r = 0.81) [62].

Zip code-level data on a median household income (scaled by $10,000) and education level (defined as a percentage of people aged 25+ who attained an educational level higher than bachelor’s degree) were obtained from the 2010–2015 American Community Survey (ACS). County level data on the numbers of primary care providers (per 100,000 residents) and the percent of uninsured individuals aged younger than 65 (supposing individuals aged 65+ are covered by the Medicare) were obtained from the Area Health Resources Files (AHRF) for 2008 and 2010–2015. County level data on prevalence of current smokers (as the percentage of the adult population in a county who reported that they currently smoke every day or most days) in age-specific groups were obtained from the Behavioral Risk Factor Surveillance System (BRFSS, CDC) for 2008–2015. County-level data are used for area-specific characteristic of behavioral (smoking) and health care related (health insurance) determinants that could impact health outcomes for studied disease.

We also included the data on As level in 5 cm of top soil in NC: As levels (in mg/kg) were obtained for 83 sites in NC from the United States Geological Survey (USGS) datafiles (10 categories for As level in top soil were used, a medium value of each range was used in the analysis) [63]. We added this variable because NC is characterized by elevated levels of naturally occurred As in groundwater and rocks of the Carolina Slate Belt [51–53] and exposure to higher levels of As has been shown to be associated with increased risk of AD and other neurodegenerative diseases [16, 52, 53].

### 2.2. Methods

The Study group includes NC populations aged 65+ living in zip codes with ambient PM_2.5_ level exceeding 10 μg/m^3^ (the WHO standard for annual PM_2.5_ concentration). The Control group includes populations living in NC zip codes with the lower 10^th^ percentile of ambient PM_2.5_ levels (≤7.61 μg/m^3^). Information on potential health effects of long-term exposures to relatively low doses of PM_2.5_ is important because of a large population size living in the US (and worldwide) being exposed to the low and moderate levels of ambient PM.

#### 2.2.1. Data collection

Mortality data were obtained from the SCHS, Vital Statistics Death, the University of North Carolina (UNC) Dataverse [64, 65]. Data on hospital admissions were obtained from the HCUP datafiles in accordance with their procedure of data request. The data on ambient PM_2.5_ levels were derived from the NASA datafiles. Data on socioeconomic characteristics and smoking were obtained from the BRFSS, the ACS, and the AHRF. The data on As levels were obtained from the US Geological Survey datafiles. More details on these data sources are provided in Data Availability section.

#### 2.2.2. Outcomes

Disease-specific mortality and hospital admissions were analyzed in the Study and Control groups for underlying causes of death and primary diagnoses of hospital admissions. To account for the situations when AD, non-AD dementia, or PD presented in patients as comorbid conditions, mortality analyses were also performed for multiple causes of death (underlying+nonunderlying causes) and hospital admissions analyses were performed for either primary or secondary diagnoses at admission. These causes were counted out from the twelve diagnoses in medical records for every case of death or hospital admission.

#### 2.2.3. Calculation of PM_2.5_ levels

To calculate PM_2.5_ levels in zip codes based on satellite values with 1.1 km resolution, an average value of the 1.1 km grid can be calculated in each zip code. In this approach we need to assume that population of each zip code gets the effect of (exposure to) an average value of PM_2.5_ in this zip code. However, the use of this approach will result in a bias due to uneven distribution of populations in zip codes, resulting in different locations of the centers of population density in predominantly urban vs. predominantly rural zip codes [51–53]. Zip codes in the Study group fall predominantly in urban Charlotte area, while zip codes in the Control group include both urban and rural areas. An example of a bias occurring when using the average value in each zip code in NC southern Piedmont area is that within this approach higher levels of PM_2.5_ caused by a wildfire near the South Carolina state border could be artificially assigned to all residents of respective zip codes. However, wildfire predominantly occurs in the areas with low population density, and using the approach of averaged levels of PM_2.5_ will result in much higher numbers than actual estimates of exposure to PM_2.5_ for populations associated with the center of zip code.

**Fig 1:**
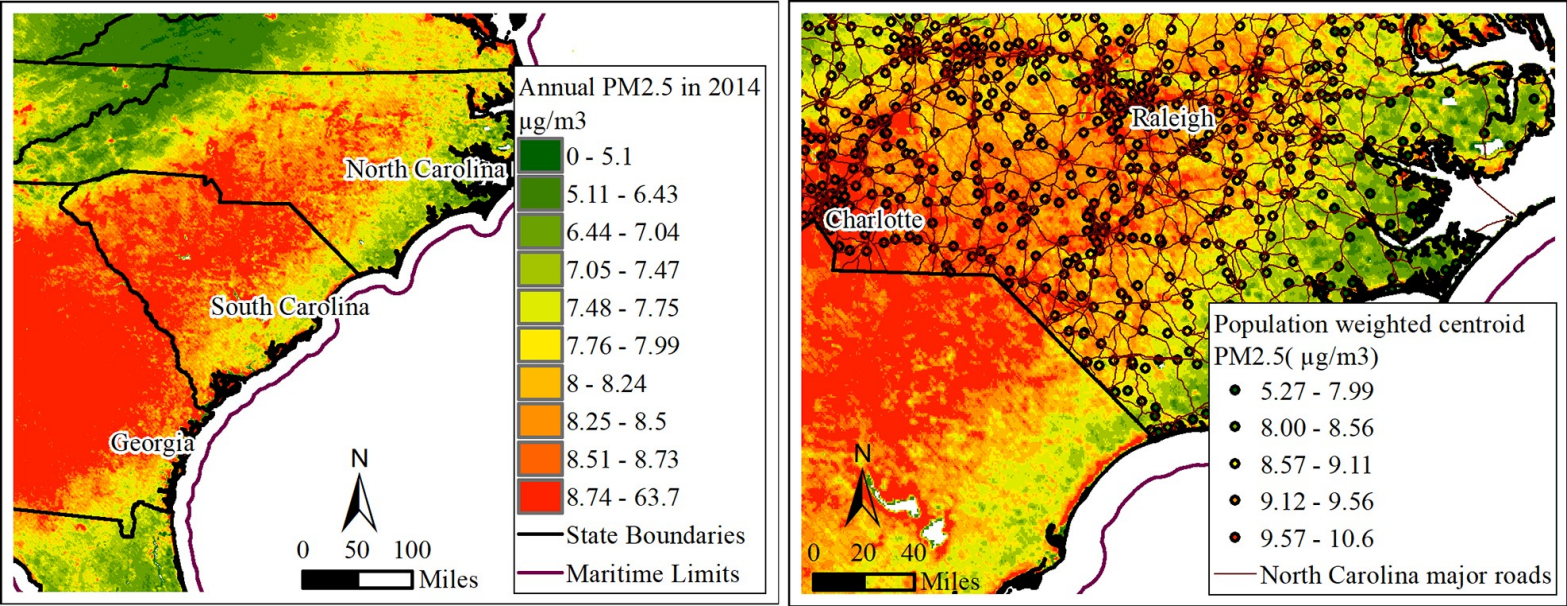
Annual concentrations of PM_2.5_ (μg/m^3^). The measures from the ground level monitors (**Fig 1A**, on the left) and the measures recalculated for the population-weighted centroids of zip codes (**Fig 1B**, on the right), North Carolina, 2014.

To minimize such bias, another approach was used in the main analysis, in which the exposure of PM_2.5_ was evaluated around population-weighted centroids. The advantage of this approach is in capturing the impact of traffic-related pollution (one of important sources of air pollution in NC), because population-weighted centroids of zip codes are located along of the major roads. Information on the centers of populations by census block groups was obtained from the US Census 2010 data. The measures for several census block groups were agglomerated into the measure for respective zip code, and populations of census block groups were used for the weights in these calculations. We set a 0.98 square mile distance around population-weighted centroids (that is equivalent 2.54 km^2^) as the 75th percentile in map size of perceived neighborhood by digitizing GIS maps [66]. The annual levels of ground monitoring of PM_2.5_ and PM_2.5_ levels in population-weighted centroids of zip codes that are mainly located along major roads are shown in (Fig 1A and 1B, respectively). As to a long-term exposure of populations to PM_2.5_ in our study, using annual or multi-year average concentrations as surrogates for a long-term air pollutant exposure is a common practice in epidemiologic studies because of highly correlated year-to-year pollutant levels [67, 68].

#### 2.2.4. Statistical analysis

First, we empirically estimated age-adjusted rates of mortality and hospital admissions per 100,000 population. The 2000 U.S. Standard Population (Census P25-1130) was used for calculating age-adjusted rates. The standard errors (SEs) and 95% confidence intervals (CIs) for the age-adjusted rates were estimated based on the approximation suggested by Keyfitz [69]: $SE=\mathrm{rate}/\sqrt{n}$, where *n* is the total number of events (deaths or hospital admissions for disease of interest), and *CI* = *rate*±1.96⋅*SE*. To avoid a potential bias due to uncertainties in population counts in NC zip codes over the study period, the odds ratios (ORs) for AD, non-AD dementia, and PD with the Control group being a reference group were additionally studied instead of respective rates/hazard ratios. In this approach, the ORs were estimated using the logistic regression model applied to the case-only datasets (both for mortality and hospital admissions) and, therefore, this approach was free from uncertainties from poorly known population counts in certain zip codes. Specifically, the following model was applied: $\mathrm{logit}(p)=u+\beta \cdot PM_{2.5}+\sum_{i}\beta_{i}o_{i}$, where *p* is the probability that an individual record in the case-only datasets is associated with disease of interest, *β* = *log*(*OR*) describes the effect of PM_2.5_ on the disease, and the term $\sum_{i}\beta_{i}o_{i}$ corresponds to other predictions that are added in the multivariable model. The estimated ORs are related to the risk ratio and several other well interpretable measures (see S1 File).

In our analyses, ORs were adjusted by age, race, sex, median household income, education, health insurance coverage, numbers of primary care providers, smoking prevalence, and level of As in the top soils. SAS Proc Logistic (the SAS 9.4 statistical package; SAS Institute, Cary, NC) was used to evaluate ORs, 95% CIs, and p-values. The Bonferroni correction was applied to adjust for multiple comparisons.

#### 2.2.5. Sensitivity analysis

In the sensitivity analysis, we assumed that population in each zip code has been exposed to an average value of PM_2.5_ in this zip code; therefore, age-adjusted rates, ORs of mortality, and ORs of hospital admissions were calculated using the data on averaged ambient PM_2.5_ levels. The results of sensitivity analysis were compared to the results of the main analysis (in which the exposure to PM_2.5_ was evaluated for population-weighted centroids) to investigate how potential bias due to uneven distribution of populations in the studied NC counties can impact the associations between PM_2.5_ levels and studied health incomes.

Additional sensitivity analysis was performed using population-weighted centroids approach (described for the main analysis in Methods section) by applying it to exposures to various levels of PM_2.5_: specifically, while in the main analysis the Study group was defined by PM_2.5_ level exceeding the WHO standard (10 μg/m^3^), in the sensitivity analysis we studied NC zip codes with PM_2.5_ levels ≥9.59 μg/m^3^(the 75^th^ percentile of ambient PM concentrations), ≥8.97 μg/m^3^(the 50^th^ percentile), ≤8.31 μg/m^3^ (the 25^th^ percentile), and ≤7.61 μg/m^3^(the 10^th^ percentile).

#### 2.2.6. Ethics approval

This is a secondary data analysis. All data analyses were designed and performed in accordance to ethical standards of the responsible committee on human studies and with the Declaration of Helsinki (of 1975, revised in 2013) and have been approved by the Duke University Health System Institutional Review Board for Clinical Investigations (IRB FWA00009025).

## 3. Results

There were 87 zip codes in the Study group and 81 zip codes in the Control group with 1,665,073 and 357,574 person-years of observation of population aged 65+, respectively (Table 1). The percent of population aged 65+ in the Study group was lower than in the Control group: 10.8% vs. 16.1% (p<0.001). This difference is in a large extent due to demographic characteristics of the Mecklenburg county that includes Charlotte urban area representing substantial part of the population in the Study group. Currently, the population in this area is younger than in the rest of NC; however, the proportion of individuals aged 65+ (and, as a result, the prevalence of aging-related diseases) is projected to grow substantially in this area in the next decades [70]. The average level of ambient PM_2.5_ in the Study group exceeded the level in the Control group: 10.27 μg/m^3^ vs. 6.92 μg/m^3^, p<0.001 (Table 1). Compared to the Control group, population in the Study group had a higher percent of African-American and a lower percent of White residents, more individuals with bachelor or higher degree, and higher rates of primary care providers (Table 1). The level of As in the top soil in the Study group was 4.41 mg/kg vs. 3.05 mg/kg in the Control group (p<0.001).

**Table 1:** Descriptive table of the studied populations: NC, 2007–2014.

Characteristics	Study group^1^	Control group^2^
Average ground level of PM_2.5_, μg/m^3^	10.27**	6.92
Population aged 65+, person-years	1,665,073	357,574
Gender, age 65+: Females	58.9%	55.0%
Race, age 65+: White	75.8%**	89.9%
African-American	20.5%**	8.8%
Population aged 65+, % of the total population	10.8%**	16.1%
Number of primary care providers (per 100,000)	100.2**	64.2
Smokers prevalence, % among aged 24+	23.3%	23.2%
Uninsured individuals, % among <65 years old	18.1%*	19.3%
Median household income, US dollars	$50,195	$45,230
Individuals with bachelor or higher degree, % among aged 25+	30.9%*	23.1%
Arsenic concentration in top soils, mg/kg	4.14**	3.06

Note

^1^87 zip codes with PM_2.5_ level ≥10 μg/m^3^

^2^81 zip codes with PM_2.5_ level ≤7.61μg/m^3^

* - 0.001<p<0.05

** -p<0.001.

Age-adjusted rates of mortality and hospital admissions for AD, non-AD dementia, and PD were higher in the Study group (Table 2), with the most pronounced between-the-groups differences observed for AD mortality (323/100,000 vs. 257/100,000 in the Study vs. Control group). In the Study group, higher mortality rates were observed among White than among African-American residents: for AD (349/100,000 vs. 236/100,000), non-AD dementia (627/100,000 vs. 574/100,000), and PD (96.1/100,000 vs. 33.9/100,000).

**Table 2:** Age-adjusted rates of mortality and hospital admissions in the study and Control groups per 100,000: Race-specific analysis, underlying causes of death/primary diagnoses.

Disease	Outcome	All races		White		African-American	
		Study group^1^	Control group^2^	Study group	Control group	Study group	Control group
Alzheimer’s disease	Mortality	323*	257	349*^a^	258	236	240
		(314–331)^3^	(240–274)	(338–360)	(239–278)	(220–253)	(189–292)
	Hospital admissions	1180*	877	1022*	641^b^	983*	824
		(1163–1196)	(845–908)	(1003–1041)	(610–672)	(949–1017)	(727–920)
Non-AD dementia	Mortality	609*	565	627* ^a^	591 ^b^	574	515
		(598–621)	(540–591)	(612–641)	(561–620)	(548–600)	(441–590)
	Hospital admissions	4346*	3177	3739* ^a^	2589 ^b^	4355*	3274
		(4314–4378)	(3117–3236)	(3703–3775)	(2527–2651)	(4283–4427)	(3081–3468)
Parkinson’s disease	Mortality	82.3*	72.9	96.1* ^a^	80.8 ^b^	33.9	20.1
		(77.9–86.7)	(63.9–81.9)	(90.3–101.9)	(69.9–91.8)	(27.6–40.3)	(5.2–35.0)
	Hospital admissions	488*	390	483* ^a^	315 ^b^	208	172
		(477–499)	(369–411)	(470–496)	(293–336)	(192–223)	(127–217)

Notes

^1^87 zip codes with PM_2.5_ level ≥10 μg/m^3^

^2^81 zip codes with PM_2.5_ level ≤7.61μg/m^3^; ^3^ 95% Confidence interval (CI) is shown in parentheses

*significant difference between the Study group and Control group, p<0.05

^a^ significant difference between White and African-American populations in the Study group, p<0.05

^b^ significant difference between White and African-American populations in the Control group, p<0.05.

In the Study group, death OR for AD was higher than for non-AD dementia or PD (Table 3): 1.35, 95%CI[1.24–1.48] vs. 0.97, 95%CI[0.90–1.04] and 1.13, 95%CI[0.92–1.31], respectively. These patterns were observed for underlying and for multiple causes of death. When analyzed as a primary case of hospitalization, only OR for AD was significantly increased (1.54, 95%CI[1.31–1.82]). The small differences between ORs obtained in univariable and multivariable analyses allow to speculate that the bias from potential uncertainty in cofactors measures is minor. The ORs remained significant under Bonferroni correction (Table 3).

**Table 3:** Odds ratios (ORs) of death and hospital admissions in the study group^1^.

Disease	Outcome	Type of analysis	Underlying cause/Primary diagnosis		Either primary or secondary cause^2^	
			OR	95% CI^3^	OR	95% CI
Alzheimer’s Disease	Death	Univariable	1.39^#^	1.28–1.51	1.23^#^	1.14–1.32
		Multivariable^4^	1.35^#^	1.24–1.48	1.21^#^	1.12–1.31
	Hospital admissions	Univariable	1.60^#^	1.37–1.86	1.38^#^	1.31–1.44
		Multivariable	1.54^#^	1.31–1.82	1.39^#^	1.32–1.47
Non-AD Dementia	Death	Univariable	1.07	1.01–1.15	1.05	1.00–1.11
		Multivariable	0.97	0.90–1.04	1.00	0.98–1.02
	Hospital admissions	Univariable	1.06	0.92–1.23	1.39^#^	1.35–1.42
		Multivariable	1.02	0.87–1.19	1.36^#^	1.32–1.40
Parkinson’s Disease	Death	Univariable	1.12	0.94–1.35	1.06	0.93–1.21
		Multivariable	1.13	0.92–1.31	1.10	0.95–1.27
	Hospital admissions	Univariable	1.25	0.90–1.72	1.26^#^	1.18–1.35
		Multivariable	1.14	0.81–1.61	1.33^#^	1.24–1.44

Notes

^1^87 zip codes with PM_2.5_ level ≥10 μg/m^3^; the Control group is e reference group

^2^counted out from twelve diagnoses in medical records for each death or hospital admission

^3^ 95% Confidence interval

^4^multivariable analysis is adjusted by age, race, sex, income, education, health insurance, smoking prevalence, number of primary care providers, and arsenic concentration (in the 5 cm top soils)

^#^remains significant under Bonferroni correction.

Death OR for AD was higher in White (OR = 1.31, 95%CI[1.20–1.43]) than in African-American (OR = 0.98, 95%CI[0.79–1.22]) population of the Study group (Table 4). African-American patients had higher hospitalization OR for non-AD dementia (OR = 1.64, 95%CI[1.52–1.76]). The results of race-specific analysis remained significant under Bonferroni correction.

**Table 4:** Odds ratios (ORs) of death and hospital admissions in White and African-American populations^1^, either as primary or secondary cause^2^, multivariable analysis^3^.

Disease	Outcome	White		African-American	
		OR	95% CI^4^	OR	95% CI
Alzheimer’s Disease	Death	1.31^#^	1.20–1.43	0.98	0.79–1.22
	Hospital admissions	1.41^#^	1.33–1.49	1.39^#^	1.22–1.58
Non-AD Dementia	Death	0.99	0.94–1.05	1.12	0.94–1.34
	Hospital admissions	1.33^#^	1.29–1.37	1.64^#^	1.52–1.76
Parkinson’s Disease	Death	1.06	0.90–1.24	1.71	0.77–3.78
	Hospital admissions	1.31^#^	1.21–1.42	1.21	0.91–1.61

Notes

^1^87 zip codes with PM_2.5_ level ≥10 μg/m^3^; the Control group is a reference group

^2^counted out from twelve diagnoses in medical records for each death or hospital admission

^3^multivariable analysis is adjusted by age, race, sex, income, education, health insurance, smoking prevalence, number of primary care providers, and arsenic concentration (in the 5 cm top soils)

^4^ 95% Confidence interval

^#^remains significant under Bonferroni correction.

### 3.1. Sensitivity analysis

Age-adjusted rates, as well as the ORs of mortality and hospital admissions obtained from the approach on averaging PM_2.5_ levels described in Methods section are shown in S1 and S2 Tables in S1 File. These results confirmed the direction and strength of associations obtained in the main analysis, with disease-specific age-adjusted mortality rates and death ORs being slightly higher in the sensitivity analysis as compared to the main analysis. That could be explained, at least in part, by urban/rural differences in the rates of AD, non-AD dementia, and PD in NC (further discussed in Discussion section).

The results of the analysis of age-adjusted rates of mortality and hospital admissions in populations living in NC zip codes with different levels of PM_2.5_ are shown in S3 Table in S1 File: namely, for populations living in zip codes with PM_2.5_ levels ≥9.59 μg/m^3^, ≥8.97 μg/m^3^, ≤8.31 μg/m^3^, and ≤7.61 μg/m^3^. This analysis showed a tendency to gradually increasing rates of mortality and hospital admissions with the higher levels of PM_2.5_ (for populations living in zip codes with exposures to ambient PM_2.5_ levels).

## 4. Discussion

While overall reduction of ambient air pollution in NC since the passage of the Clean Smokestacks Act (in 2001) contributed to decreases in respiratory, cardiovascular, and cerebrovascular morbidity and mortality in the state [54, 55], there remain region-specific variations in air quality. In this study, we tested the hypothesis that residents of the southern Piedmont area of NC chronically exposed to ambient PM_2.5_ levels exceeding the WHO standard for air quality (10 μg/m^3^) have higher rates of mortality and hospital admissions for AD, non-AD dementia, and PD than NC residents exposed to PM_2.5_ at the levels below the WHO standard. Our results showed a higher mortality and hospital admission rates—especially for AD—in the southern Piedmont area of NC. Other studies showed that long-term exposure to PM_2.5_ was associated with a higher risk of cognitive impairment [44, 71–73], even when the levels of ambient pollutants were relatively low [74]. Long-term exposures to relatively low levels of PM, including PM_2.5_, are experienced by many US residents; therefore, PM may substantially impact neurodegenerative disease burden across the country. Moreover, long-term exposures to PM has been shown to accelerate aging-related cognitive decline in human population: the effect of a 10μg/m^3^ increment in PM_2.5–10_ and PM_2.5_ levels can be cognitively equivalent to aging by approximately 2 years of age [74].

We found that the effects of long-term exposure to low-dose ambient PM_2.5_ on mortality and hospital admissions were highly significant for AD and less pronounced for non-AD dementia and PD. These findings persisted after an adjustment for cofactors, and when analyzed with the studied disease as underlying cause of death as well as when analyzed either for primary or secondary causes. Our findings are in general agreement with a recent meta-analysis of AD risk associated with PM exposure which demonstrated a strong association between long-term exposure to PM_2.5_ (PM levels ranging from 2.84 μg/m^3^ to 100 μg/m^3^) and AD risk (HR = 3.26, 95%CI[0.84–12.74]). The impact of PM on the risk of PD (HR = 1.34, 95%CI[1.04–1.73]) and dementia (HR = 1.16, 95%CI[1.07–1.26]) were less pronounced [47]. Association studies often focus on disease incidence [27, 47, 48, 75, 76] in contrast to mortality or hospital admissions, so, these results are not directly comparable to ours. Nonetheless, we can recalculate our results to allow some comparisons: for example, Table 3 could be recalculated per 1 μg/m^3^ increase in PM_2.5_ levels resulting in hospital admission OR’ = 1.10 (95%CI[1.09–1.12]) for AD, OR’ = 1.10 (95%CI[1.09–1.11]) for non-AD dementia, and OR’ = 1.09 (95%CI[1.07–1.12]) for PD (in multivariable analysis, for either primary or secondary cause of hospitalization). Our results are in a general agreement with the results of the study on hospital admissions of Medicare enrollees (aged 65+) in 50 northeastern US cities (note, this study did not include geographic area of our study) [39]: the levels of PM_2.5_ in 50 cities study varied from 8.21 μg/m^3^ to 15.49 μg/m^3^ and they were comparable with the levels in our Study group, and HR per 1 μg/m^3^ increase of PM_2.5_ levels was 1.15 for AD (95%CI[1.11–1.19]), 1.08 for non-AD dementia (95%CI[1.05–1.11]), and 1.08 for PD (95%CI[1.04–1.12]). Another study of 121 US cities estimated the risk of cause-specific emergency hospitalization in association with a short-term exposure to PM_2.5_ [77]. Although this study has found significant effects of PM_2.5_ on hospitalization for PD and for AD in persons aged 65–75 [77], the results of this study are not directly comparable to our results because of the differences in regression models used in analysis, period of observation, death certificate based mortality vs. mortality by previous cause of emergency hospitalization, set of cofactors, and exposure type (long-term vs. short-term).

Results from studies analyzing multiple diseases within the same cohort can provide important information of disease-specific differences resulting from PM exposure, but these studies infrequently include neurodegenerative diseases. One study of younger individuals of Mexico City has suggested air pollution being a risk factor for both AD and PD [22]: the higher risk was based on analyses of mRNA cyclooxygenase-2, interleukin-1β, and CD14 in target brain regions. Another population-based cohort study on adults aged 55–85 years in Ontario (Canada) has demonstrated that people living close to roadways had increased risk of dementia but not PD [27]. Both these studies focused on disease incidence. The abovementioned study of 50 US cities [39] showed the highest risk of hospital admissions for AD, with lower impacts on dementia and PD; however, there were substantial variations in city-specific composition of PM. To the best of our knowledge, there are no other studies comparing the impact of PM exposure on mortality and/or hospital admissions for AD, non-AD dementia, and PD in the US within one study design.

The biologic basis of a relationship between AD and PM exposures has been investigated in both laboratory and epidemiologic studies. Among the mechanisms that have been suggested are gene-environment interactions affecting ε4 allele of the apolipoprotein E (APOE) [78], triggering inflammation in the brain [79] resulting in accumulation of amyloid-beta peptide (Aβx-42 peptide, an important early biomarker of AD) [80]. In addition, dysfunction of the BBB, neural degeneration, and apoptosis in glial cells [81–83] have been observed. However, these mechanisms as well as the abovementioned meta-analysis have been described for much higher levels of PM_2.5_ than in our study.

Compared to AD studies, analyses of the association of non-AD dementia with air pollution [84] are less frequent, but some reports link dementia risk to long-term exposure [75]. In our study, an increased risk of non-AD dementia as either primary or secondary cause of hospitalization was found, but not for a primary cause. Further research is needed to determine if there is an association between the risk of non-AD dementia death or hospitalization and ambient PM_2.5_ [61]. Results obtained in our study for PD were similar to non-AD dementia patterns (Table 3): an increased risk of PD being either primary or secondary cause of hospital admission was found, but not for PD being a primary cause. While ambient PM_2.5_ has been reported to alter α-synuclein aggregation and lead to neuronal injury and the loss of dopaminergic neurons in substantia nigra [85–87] thus contributing to the development and progression of PD [88, 89], population-based studies do not provide a clear association with the risk of PD. For example, the six-city study [48] and the studies on hospitalization for PD [39, 77] reported an increased risk of PD with higher levels of PM_2.5_, but other studies reported either modest increase in risk [27] or lack of associations between PM levels and PD risk [76, 90–93], including a lack of association between PM_2.5_ levels and PD risk among NC farmers [76].

Race-specific analysis in our study showed that mortality rates from AD (349/100,000 vs. 236/100,000), non-AD dementia (627/100,000 vs. 574/100,000), and PD (96.1/100,000 vs. 33.9/100,000) were higher among White than African-American residents living in the southern Piedmont area of NC (Table 2). The AD death OR was also increased in White (OR = 1.31, 95%CI[1.20–1.43]) but not in African-American (OR = 0.98, 95%CI[0.79–1.22]) residents of this geographic area, while African-American patients had higher OR of hospitalization for non-AD dementia than their White counterparts (Table 4). These findings support the results of other studies reporting higher AD and dementia incidence rates and prevalence in African-American than White older US adults [94–96] and higher mortality rates from AD among older White populations [96–98] or between-the-races geographic variations in AD and dementia mortality along the Atlantic coast [99]. At present, explanations for greater survival after diagnosis of AD in African-Americans, if true, are lacking [100]. Further research is needed to assess the factors such as the artifacts of certification, approaches to diagnosis and access to medical care, causes of biological variations in disease progression, behavioral risks, and impacts of environmental exposures as possible causes of race-specific variations in AD and non-AD dementia morbidity and mortality [99].

The results of our study are innovative in several aspects. First, the study design allows for regional comparisons that highlights the potential impact of PM_2.5_ levels which only slightly exceed the WHO standards. Information for this range of air contaminants is important because of a large percentage of the population in the US and worldwide being exposed to low and moderately increased levels of ambient PM. Appreciating the health impacts of low and moderate levels of PM highlight the need to address the regional differences in air contaminants to reduce the risk of neurodegenerative diseases in at-risk populations. The second innovation is that age, race, sex, income, education, health insurance, smoking prevalence, number of primary care providers, and As concentrations were considered as cofactors within the same population and geographic locations. Previous studies either failed to adjust for detailed set of cofactors or made crude adjustments for sociodemographic factors [34, 40, 44, 101, 102]. At our best knowledge, no other studies of this type have included As in the set of cofactors. Inclusion of As in our analysis was motivated by the fact that the central-south NC (including the southern Piedmont area) has elevated levels of naturally occurred As in the Carolina Slate Belt [51–53]. Another important fact about As is that exposure to higher levels of As has been shown to be associated with increased risk of AD and other neurodegenerative diseases [16, 52, 53]. It has been suggested that As could impact neurodegeneration through the mechanisms of activation of the p38 MAPK and JNK3 with neuronal apoptosis [52] and affect an induction of hyperphosphorylation of protein tau and over-transcription of the amyloid precursor protein involved in the formation of neurofibrillary tangles and brain amyloid plaques [53]. Third, the size of population in our study is relatively large and it is ascertained from validated registries that enabled us to examine the health effects for over an eight-year period. Finally, the results provide an insight on contribution of ambient PM_2.5_ to the decades of increased AD mortality rates in southern Piedmont area in NC: at present, the studies focused on populations living in this area remain sparse, while the observed poorer AD outcomes persist.

### 4.1. Discussion on sensitivity analysis

Age-adjusted mortality rates and death ORs were slightly higher than in the main analysis. That could be explained, at least in part, by urban/rural differences in neurodegenerative disease rates in NC. While death rates of AD in the US are lower among the residents of urban areas [103], the urban/rural differences substantially varies from state to state. In many US states, mortality from AD does not differ between urban and rural areas and in some states (e.g. Arizona, California, Colorado, and North Carolina) AD mortality is higher in urban than in rural areas (up to 26%-80%, based on 2007–2014 data for population aged 65+) [104]. Therefore, slightly higher rates and ORs in sensitivity analysis could be due to more strict division between urban (predominantly in the Study group) and rural (predominantly in the Control group) zip codes with higher death rates in NC urban areas (as mentioned above, this is a unique characteristic of NC [104]).

### 4.2. Study limitations

This study has limitations that are common for observational studies due to ingrained biases such as information bias and selection bias. These limitations include the absence of an individual exposure and a history of the residence for a prolonged period of time, not accounting for traffic noise as potential risk factor of AD and dementia, and not accounting for variations in exposure to PM within a small geographical area (areas that are smaller than zip code level). Due to a lack of individual-level information on socioeconomic characteristics and behavioral factors, we adjusted for these factors on zip code and county levels, since the neighborhood socioeconomic status is strongly associated with individual socioeconomic status and behavioral variables [105, 106]. Possible misclassification between AD and non-AD dementia is another limitation of this data analysis. However, based on the directions of associations obtained for these diseases we do not expect substantial impact of misclassification between these two diagnoses on the conclusion obtained from this study. Finally, we reported estimations for a single ambient pollutant (PM_2.5_) without considering detailed analysis of the components (e.g., metals) in PM_2.5_. This is an important direction of future studies, because the spectrum of metals and their concentrations in PM_2.5_ are specific for certain geographic areas [107–109] and specific components are associated with specific disease risks (e.g., increased risk of PD was associated with higher levels of manganese, copper, or mercury in the air [110, 111]).

## 5. Conclusion

The brain has been identified as a critical target of air pollution, in particular small particulates which are associated with increased risk of neurodegenerative diseases. Regional variations in air quality enable us to test the hypothesis that residents chronically exposed to elevated levels of ambient PM_2.5_ have a greater risk of death or hospital admissions due to neurodegenerative diseases. The rates of mortality and hospital admission risks for AD, and in lesser extent for non-AD dementia and PD were found for the residents of the southern Piedmont area of NC exposed to ambient PM_2.5_ levels that exceed WHO standard. In addition, racial disparities in these associations were observed: exposures to PM_2.5_ were strongly associated with mortality in White and with hospitalization in African-American populations. Biomedical, socioeconomic, and environment-related mechanisms behind these disparities require further analyses. Since the opportunity of an individual to control the risk from air pollution is low [112], further information about regional variations in air quality and further regulations of public exposure to air pollutants (e.g., modifications addressing the energy production and traffic patterns in the southern Piedmont area) could provide a great potential for improving neurocognitive health in at risk US populations.

## Supporting information

S1 File (DOCX)
