# Supplementary material for: Exposure to low-dose ambient fine particulate matter PM2.5 and Alzheimer’s disease, non-Alzheimer’s dementia, and Parkinson’s disease in North Carolina
Source: PLoS One. 2021 Jul 9;16(7):e0253253. doi: 10.1371/journal.pone.0253253 (PMC8270415; doi:10.1371/journal.pone.0253253)
Supplement: S1 File — (DOCX) [file pone.0253253.s001.docx]

**Supplementary Methodological Remark**

In our study, the ORs of deaths from a specific disease among all deaths and the ORs of hospital admissions for a specific disease among all-cause hospital admissions are represented as:

$$OR^{d}=\frac{C_{study}^{d}}{C_{study}^{t}-C_{study}^{d}}\cdot\frac{C_{control}^{t}-C_{control}^{d}}{C_{control}^{d}}=\frac{C_{study}^{d}/P_{study}}{C_{control}^{d}/P_{control}}\cdot\frac{C_{control}^{t}/P_{control}}{C_{study}^{t}/P_{study}}\cdot\frac{1-f_{control}^{d}}{1-f_{study}^{d}}=HR^{d}\cdot\frac{1}{h}\cdot\frac{1-f_{control}^{d}}{1-f_{study}^{d}}$$

where *C* and *P* denote the numbers of disease-specific cases (deaths or hospital admissions) in the Study and the Control groups and disease-independent person-years, respectively; super-indices “*d*” and “*t*” show disease-specific and total (i.e., all-cause) measures, respectively;

Thus, the ORs (estimated using the case only dataset) are expressed as a product of three terms: 1) disease-specific hazard ratio, $HR^{d}$, i.e., the ratio of the hazard functions in the Study and the Control groups; 2) disease-independent hazard ratio of the total hazards, $h$, in two groups; and 3) the factor containing the frequencies ($\approx1-(f_{control}^{d}-f_{study}^{d})$ ) that deviates from one by the difference in frequencies in two groups and could be negligible for majority of diseases.

This decomposition allows us to connect our OR to HR and several other clearly interpretable measures (e.g., listed above #2 and #3) and is kept in mind when interpreting the study estimates. Improved accuracy in estimation of OR that avoid the use any population estimates overweight the slightly more complex ways of interpretation.

**Supplementary Tables**

**S1 Table.** Age-adjusted rates of outcomes obtained from approach on averaging PM_2.5_ levels, North Carolina, 2007-2014.

| Disease | Outcome | Study group^1^ | Control group^2^ |
| --- | --- | --- | --- |
| Alzheimer’s disease | Mortality | 392* (379-404)^3^ | 262 (252-270) |
|  | Hospital admissions | 1242* (1220-1265) | 814 (800-828) |
| Non-AD dementia | Mortality | 557* (542-572) | 582 (570-594) |
|  | Hospital admissions | 4423* (4380-4466) | 3024 (2997-3052) |
| Parkinson’s disease | Mortality | 81.6 (75.8-87.6) | 76.4 (72.0-80.8) |
|  | Hospital admissions | 451* (437-465) | 370 (361-380) |

Notes: ^1^87 zip codes with PM_2.5_ level ≥10 µg/m^3^; ^2^81 zip codes with PM_2.5_ level ≤7.61µg/m^3^; ^3^the 95% CIs are shown in parentheses; ^*^significant difference between the groups, p<0.05.

**S2 Table.** Odds ratios (ORs) of death and hospital admissions obtained in approach of averaging PM_2.5_ levels^1^, North Carolina, 2007-2014.

| Disease | Outcome | Type of analysis | Underlying cause/Primary diagnosis | | Either primary or secondary cause^2^ | |
| --- | --- | --- | --- | --- | --- | --- |
|  |  |  | OR | p value | OR | p value |
| Alzheimer’s | Mortality | Univariable | 1.63^#^ | <0.0001 | 1.49^#^ | <0.0001 |
| disease |  | Multivariable^3^ | 1.43^#^ | <0.0001 | 1.31^#^ | <0.0001 |
|  | Hospital | Univariable | 1.52^#^ | <0.0001 | 1.51^#^ | <0.0001 |
|  | admissions | Multivariable | 1.15 | 0.1234 | 1.44^#^ | <0.0001 |
| Non-AD | Mortality | Univariable | 1.08 | 0.0310 | 0.97 | 0.2292 |
| dementia |  | Multivariable | 0.94 | 0.1529 | 0.94 | 0.0828 |
|  | Hospital | Univariable | 1.05 | 0.5419 | 1.43^#^ | <0.0001 |
|  | admissions | Multivariable | 0.98 | 0.8284 | 1.43^#^ | <0.0001 |
| Parkinson’s | Mortality | Univariable | 1.19 | 0.0406 | 1.07 | 0.3096 |
| disease |  | Multivariable | 1.26 | 0.0337 | 1.20 | 0.0342 |
|  | Hospital | Univariable | 1.03 | 0.8342 | 1.18^#^ | <0.0001 |
|  | admissions | Multivariable | 0.76 | 0.1561 | 1.22^#^ | <0.0001 |

Notes: ^1^87 zip codes with PM_2.5_ level ≥10 µg/m^3^; the Control group is a reference group; ^2^counted out from twelve diagnoses in medical records for each death or hospital admission; ^3^multivariable analysis is adjusted by age, race, sex, income, education, health insurance, smoking prevalence, number of primary care providers, and arsenic concentration (in the 5 cm top soils); ^#^remains significant under Bonferroni correction.

**S3 Table.** Age-adjusted rates of mortality and hospital admissions in populations living in zip codes with different levels of PM_2.5_ levels across North Carolina, 2007-2014.

| Age-adjusted rate | Study group  (PM_2.5_ level  ≥10 μg/m^3^) | 75^th^ percentile  (PM_2.5_ level  ≥9.59 μg/m^3^) | 50^th^ percentile  (PM_2.5_ level  ≥8.97 μg/m^3^) | 25^th^ percentile  (PM_2.5_ level  ≤8.31 μg/m^3^) | 10^th^ percentile  (PM_2.5_ level  ≤7.61 μg/m^3^) | 5^th^ percentile  (PM_2.5_ level  ≤7.28 μg/m^3^) |
| --- | --- | --- | --- | --- | --- | --- |
| Alzheimer’s disease: Mortality | 322.8  (314.2-331.4)^1^ | 314.8  (309.1,320.4) | 301.9  (297.7,306.0) | 248.4  (239.7-257.0) | 256.8  (239.9-273.8) | 235.8  (214.2-257.5) |
| Hospital admissions | 1180  (1163-1196) | 1196  (1185-1207) | 1145  (1137-1153) | 816.4  (800.5-832.2) | 876.7  (845.3-908.2) | 804.8  (764.4-845.2) |
| Non-AD dementia: Mortality | 609.3  (597.5-621.1) | 638.4  (630.4,646.4) | 637.7  (631.6,643.8) | 576.8  (563.5-590.0) | 565.3  (540.1-590.5) | 561.0  (527.4-594.5) |
| Hospital admissions | 4346  (4314-4378) | 4312  (4291-4333) | 4129  (4114-4145) | 3027  (2997-3057) | 3177  (3117-3236) | 3253  (3172-3334) |
| Parkinson’s disease: Mortality | 82.3  (77.9-86.7) | 83.71  (80.77,86.64) | 86.33  (84.07,88.60) | 79.2  (74.3-84.1) | 72.9  (63.9-81.9) | 70.1  (58.2-82.0) |
| Hospital admissions | 487.9  (477.1-498.7) | 475.6  (468.6-482.6) | 476.2  (470.8-481.5) | 377.9  (367.2-388.7) | 389.8  (369.0-410.7) | 376.4  (348.7-404.0) |

Notes: ^1^the 95% CIs are shown in parentheses.
